# 2559 Role of tissue non-specific alkaline phosphatase (TNAP) in promoting the survival of acute myeloid leukemia (AML) cells within the bone marrow microenvironment

**DOI:** 10.1017/cts.2018.119

**Published:** 2018-11-21

**Authors:** Bradley Bowles, Rosalie M. Sterner, Kimberly N. Kremer, Amel Dudakovic, Jennifer J. Westendorf, Andre J. Van Wijnen, Karen E. Hedin

**Affiliations:** 1 Mayo Clinic; 2 Mayo Clinic Medical Science Training Program, Mayo Clinic Department of Immunology; 3 Mayo Clinic Department of Immunology; 4 Mayo Clinic Department of Orthopedic Surgery; 5 Mayo Clinic Department of Orthopedic Surgery, Department of Biochemistry and Molecular Biology; 6 Mayo Clinic Department of Immunology

## Abstract

OBJECTIVES/SPECIFIC AIMS: Treatment of acute myeloid leukemia (AML) is challenging, as apoptosis-resistant AML cells often persist within the bone marrow microenvironment despite chemotherapy. The overall goal of our laboratory is to identify and ultimately target the bone marrow factors that protect AML cells. METHODS/STUDY POPULATION: Using cell cultures, we previously reported that SDF-1 (CXCL12), an abundant bone marrow chemokine, induces apoptosis of isolated CXCR4+ AML cells, including freshly isolated bone marrow-derived AML cells from approximately one-third of AML patients. However, co-culture of AML cells with differentiating osteoblasts protected AML cells from apoptosis. RESULTS/ANTICIPATED RESULTS: Histone deacetylase inhibitors (HDACi) abrogated the ability of osteoblasts to protect AML cells and altered expression of matrix mineralization genes including tissue nonspecific alkaline phosphatase (TNAP). A different drug, cyclosporine A (CSA), similarly inhibited osteoblast-mediated protection of AML cells and reduced TNAP expression. Specifically targeting osteoblast TNAP via siRNA was sufficient to prevent osteoblasts from protecting AML cells in co-cultures. In addition, we are targeting TNAP enzymatically. DISCUSSION/SIGNIFICANCE OF IMPACT: Our results indicate that targeting TNAP may be useful in AML treatment to render the bone marrow microenvironment more hostile to leukemic cell survival.

